# Comparative pericarp biomechanics and germination physiology of *Raphanus raphanistrum* and *Raphanus pugioniformis* indehiscent fruits

**DOI:** 10.1093/aob/mcaf015

**Published:** 2025-01-27

**Authors:** Tina Steinbrecher, Samik Bhattacharya, Jonathan Binder, Katharina Kleemeier, Felix Przesdzink, Franziska Groene, Kyra Jacoblinnert, Klaus Mummenhoff, Gerhard Leubner-Metzger

**Affiliations:** Seed Biology and Technology Group, Department of Biological Sciences, Royal Holloway University of London, TW20 0EX, Egham, UK; Department of Biology, Botany, University of Osnabrück, D-49076 Osnabrück, Germany; Resolve Biosciences, 40789 Monheim am Rhein, Germany; Seed Biology and Technology Group, Department of Biological Sciences, Royal Holloway University of London, TW20 0EX, Egham, UK; Department of Biology, Botany, University of Osnabrück, D-49076 Osnabrück, Germany; Department of Biology, Botany, University of Osnabrück, D-49076 Osnabrück, Germany; Department of Biology, Botany, University of Osnabrück, D-49076 Osnabrück, Germany; seedalive, 49076 Osnabrück, Germany; Department of Biology, Botany, University of Osnabrück, D-49076 Osnabrück, Germany; Department of Biology, Botany, University of Osnabrück, D-49076 Osnabrück, Germany; seedalive, 49076 Osnabrück, Germany; Seed Biology and Technology Group, Department of Biological Sciences, Royal Holloway University of London, TW20 0EX, Egham, UK; Laboratory of Growth Regulators, Faculty of Science, Palacký University and Institute of Experimental Botany, Czech Academy of Sciences, CZ-78371 Olomouc, Czech Republic

**Keywords:** Functional morphology, finite element stress simulation, fruit coat biomechanics, pericarp-imposed mechanical dormancy, predetermined breaking zone, soil seed bank persistence

## Abstract

**Background:**

The biomechanical, morphological and ecophysiological properties of plant seed/fruit structures are adaptations that support survival in unpredictable environments. High phenotypic variability of noxious and invasive weed species such as *Raphanus raphanistrum* (wild radish) allow diversification into new environmental niches. Dry indehiscent fruits (thick and lignified pericarp [fruit coat] enclosing seeds) have evolved many times independently.

**Methods:**

A multiscale biomechanics and imaging (microscopy, X-ray, finite element stress simulation, puncture force analysis) approach was used to comparatively investigate the indehiscent fruits of *R. raphanistrum* (global weed), *R. pugioniformis* (endemic weed) and *R. sativus* (cultivated radish).

**Results:**

The hard pericarp of *Raphanus* species (Brassicaceae) imposes mechanical dormancy by preventing full phase-II water uptake of the enclosed seeds. The apparently unilocular fruits of *Raphanus* species develop from two fused valves, pericarp rupture to permit germination is confined to the midvalve regions, and each midvalve region contains a predetermined breaking zone that is biomechanically defined by the internal shape of the seed chambers. Direct biomechanical analysis revealed great variability in within-fruit and between-fruits pericarp resistances.

**Conclusions:**

Variability in pericarp-imposed dormancy provides a bet-hedging strategy to affect soil seed bank persistence and prolong the germinability period.

## Graphical Abstract

**Graphical Abstract:**
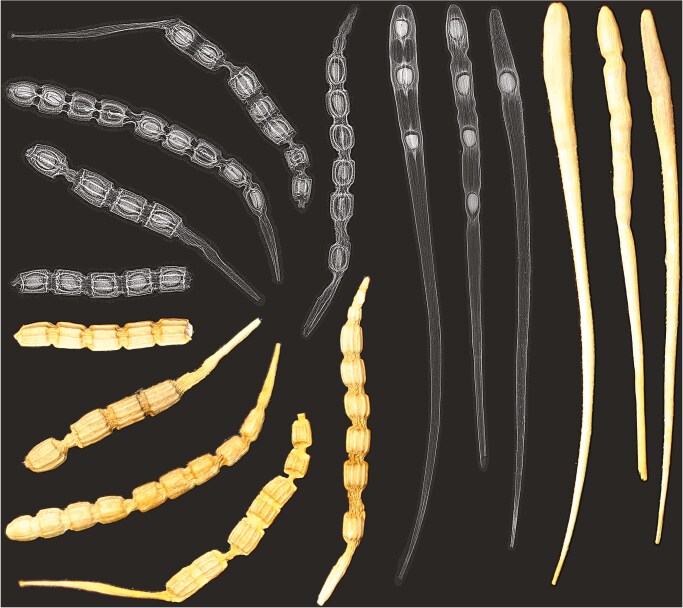

## INTRODUCTION

Dry indehiscent fruits, rather than seeds, have evolved as dispersal and germination units in many angiosperms as an adaptation to allow diversification into new environmental niches (Mummenhoff *et al.*, 2009; Dardick and Callahan, 2014; Arshad *et al.*, 2019; Bobrov and Romanov, 2019; Grafi, 2020). The pericarp (fruit coat) of dry indehiscent fruits is dead tissue, encases single or multiple seeds, and its inner layer (endocarp) is often hardened by the lignification of thick-walled cells (Sperber *et al.*, 2017; Vercellino *et al.*, 2019; Wu *et al.*, 2023). The means by which hard seed-covering layers (lignified or stony pericarps) open during germination were first investigated in 1933 by Sir Arthur Hill, Director of the Royal Botanic Gardens (Kew, London). Hill (1933) and others (Collinson *et al.*, 2012) found Eocene fossil fruits with hard endocarps. Global climate change processes in the Eocene were identified as the primary selective agents for physical dormancy characterized by water-impermeable hard seed or fruit coats (Baskin and Baskin, 2014; Steinbrecher and Leubner-Metzger, 2017, 2018). However, most ‘hard-seeded’ species have physiological dormancy or non-dormancy with water-permeable seed or fruit coats (e.g. Cousens *et al.*, 2010; Lu *et al.*, 2015, 2017*a*, *b*; Zhou *et al.*, 2015; Sperber *et al.*, 2017; Vercellino *et al.*, 2019; Hourston *et al.*, 2022; Chandler *et al.*, 2024). Dormancy is an innate seed/fruit property that defines the environmental conditions required for germination (Finch-Savage and Leubner-Metzger, 2006; Baskin and Baskin, 2014; Steinbrecher and Leubner-Metzger, 2018). It is a highly adaptive trait that also controls weed seed bank persistence and the timing of weed seedling emergence (Baskin and Baskin, 2014; Batlla *et al.*, 2020; Nakabayashi and Leubner-Metzger, 2021).

There is a vast amount of angiosperm fruit morphological diversity as adaptations in seed protection and dispersal strategies (Dardick and Callahan, 2014; Pabon-Mora *et al.*, 2014; Eldridge *et al.*, 2016; Ezra *et al.*, 2023; Lazarus *et al.*, 2023). While most Brassicaceae have dry dehiscent fruits, several species have dry indehiscent siliques or silicles as dispersal units. These are capsular fruits of different shapes and sizes derived from two fused carpels (Neya *et al.*, 2008; Cousens *et al.*, 2010; Lu *et al.*, 2015, 2017*b*; Zhou *et al.*, 2015; Sperber *et al.*, 2017; Tricault *et al.*, 2017; Mohammed *et al.*, 2019; Vercellino *et al.*, 2019; Chandler *et al.*, 2024). Their morphological and biomechanical pericarp properties are of adaptive value to ensure survival in response to the prevailing environment. Pericarp properties are associated with seed/fruit dormancy and longevity, seed/fruit persistence in the seed soil bank, and germination phenology and seasonal emergence patterns (Lu *et al.*, 2015, 2017*b*; Tricault *et al.*, 2017; Vercellino *et al.*, 2019). For dispersed dry indehiscent fruits, abiotic (temperature and water availability in the soil seed bank) and biotic (fungal release of pericarp-imposed mechanical dormancy) factors control germination timing of seeds enclosed by the pericarp (Sperber *et al.*, 2017; Tricault *et al.*, 2017; Vercellino *et al.*, 2019; Wu *et al.*, 2023), but very little is known about their underpinning mechanisms. Fruit fracture analysis demonstrated that the pericarp-imposed mechanical dormancy in *Lepidium didymum* (Brassicaceae) controls germination solely by a biomechanical mechanism (Sperber *et al.*, 2017).

*Raphanus sativus* (cultivated radish) is an important root crop in the Brassicaceae family which has dry indehiscent fruits as dispersal units and is known to also hybridize with wild radishes (Heredia and Ellstrand, 2014; Vercellino *et al.*, 2019, 2023). Its probable wild ancestor, *R. raphanistrum* (wild radish), is an invasive and noxious weed of global distribution (Kebaso *et al.*, 2020). In the East Mediterranean area, the genus *Raphanus* includes two closely related annual weed species with dry indehiscent fruits as dispersal units: *R. raphanistrum* and *R. pugioniformis* (Ziffer-Berger *et al.*, 2020; Bhattacharya *et al.*, 2022; Wasserstrom *et al.*, 2022). *Raphanus raphanistrum* is widely distributed around the Mediterranean Basin as its native range, while *R. pugioniformis* is endemic, with its distribution limited to northern Israel and southern Lebanon and Syria (Ziffer-Berger *et al.*, 2020). After detachment from the mother plant by abscission during summer, *R. raphanistrum* dry indehiscent fruits usually break into several one-seeded lightweight segments as dispersal units, whereas the relatively long and heavy *R. pugioniformis* fruits remain intact after detachment by abscission. Ziffer-Berger *et al.* (2020) concluded that these differences in fruit structure and functionality contributed to differences in dispersal ability and spatial distribution of the two wild radishes. The dry indehiscent fruits of *Raphanus* species therefore provide an excellent system (Cousens *et al.*, 2010; Heredia and Ellstrand, 2014; Tricault *et al.*, 2017; Vercellino *et al.*, 2019; Ziffer-Berger *et al.*, 2020) to investigate how the indehiscent pericarp controls dormancy and germination.

In this study, we comparatively investigated the morphological, ecophysiological and biomechanical mechanisms of the pericarp of the two wild radish weeds *R. raphanistrum* and *R. pugioniformis*. Our results reveal the distinct and conserved aspects of their pericarp morphology and function during simulated soil seed bank storage and germination of seeds enclosed by the pericarp. We found that the lignified endocarp confirms mechanical dormancy, which delays or prevents the germination of seeds enclosed by the pericarp. Pericarp rupture to permit radicle emergence from imbibed siliques was associated with a predetermined breaking zone (PBZ) in the midvalve region (MVR) of dispersed *Raphanus* fruits. Biomechanical identification and detailed analysis of PBZ functionality provided mechanistic insight into the role of the pericarp in dormancy and germination of dispersed indehiscent fruits.

## MATERIALS AND METHODS

### Plant material and weather data

Mature indehiscent fruits of *Raphanus raphanistrum* and *Raphanus pugioniformis* were collected in Israel as described (Ziffer-Berger *et al*., 2020). The *R. raphanistrum* accession (RAN) was collected in Ra’anana close to Tel Aviv (32°11′27.80″N 34°50′45.73″E) and the *R. pugioniformis* accession (YHD) in Yehudia north-east of the Sea of Galilee (32°57′10.01″N 35°42′23.02″E) (Supplementary Data Fig. S1A). Weather data across seasons in their natural habitats were obtained and are presented in Supplementary Data Fig. S1. Air-dried fruits were stored for after-ripening in the laboratory at the University of Osnabrück for at least 10 months (at ~25 °C and uncontrolled relative humidity). Enclosed seeds within intact fruits as well as isolated (bare) seeds were used in the experiments; the latter were obtained by manually removing the pericarp. The 100-seed weights, sizes and moisture and dry weight of bare seeds were obtained as described in Supplementary Data Fig. S2.

### Germination assays

Germination assays were performed using three to six replicate plates of 25 bare seeds in 60-mm Petri dishes. Seeds were imbibed with 3 mL of autoclaved deionized water (dH_2_O) on two layers of filter papers (MN713; Macherey-Nagel, Dueren, Germany) and sealed using Parafilm. The assays were performed in MLR-352 Versatile Environmental Test Chambers (PHC Europe B.V., Breda, The Netherlands). Standard germination conditions used were a day (16 h white light [100 µmol m^−2^ s^−1^] at 25 °C) and night (8 h darkness at 15 °C) cycle for usually up to 1 month. Germination assays with intact fruits were conducted under the same conditions using a total number of 24–26 fruits for each treatment containing on average five *R. raphanistrum* and three *R. pugioniformis* seeds per fruit. Completed germination was scored at least daily using a stereomicroscope7 as visible radicle protrusion, which directly succeeds testa rupture (seed) or pericarp rupture (fruit). Water uptake into seeds (three replicate plates of 25 bare seeds or at least six fruits) was conducted by weighing bare seeds versus seeds enclosed in intact fruits over time and calculating the seed moisture content per dry weight.

### Microscopy and X-ray analyses

In preparation for microscopical analysis, fruits just prior to maturity drying were fixed in FAA (5 % v/v formaldehyde, 60 % v/v ethanol, 0.1 % v/v Tween-20) first under vacuum (~100 mbar) and then at 4 °C for 48 h. Samples were subsequently washed twice with ice-cold 70 % ethanol followed by overnight dehydration in 70 % ethanol at 4 °C. These samples were further dehydrated by incubations in 85 % (1 h), 95 % (1 h) and 100 % (overnight) ethanol. The ethanol was replaced stepwise (2:1, 1:1, 1:2; 30 min each) by several hours of incubation in Histoclear (Hycultec GmbH, 94501 Beutelsbach, Germany), which was subsequently embedded in paraffin (P3683, Sigma–Aldrich, Darmstadt, Germany) by incubation at 57 °C with replacement of the paraffin solution twice a day; paraffin blocks were stored at 4 °C. Thin (8–15 µm) cross-sections were cut using an ultramicrotome (RM2125, Leica, Nussloch, Germany), and histo-stained with safranin/astra blue as described (Sperber *et al.*, 2017). A light microscope (BX43, Olympus, Hamburg, Germany) was used to reveal the anatomy and to histochemically identify lignin in the histo-stained cross-sections of *R. raphanistrum* and *R. pugioniformis* fruits as described by Sperber *et al.* (2017). X-ray imaging was conducted using a COMPAI TrueView 100Pro device at 50 kVp and 20 mAs (RPS Service Ltd, Byfleet, UK).

### Biomechanical analyses and finite element modelling

Biomechanical analysis was carried out using a ZwickRoell universal testing machine (ZwickiLine Z0.5) with a custom force sensor (ZwickRoell Group, Ulm, Germany). To determine the puncture force of the pericarp of individual fruit segments with one seed chamber, the segments were cut in half either along the MVR or perpendicular to the MVR and the seed was removed from the seed chamber. Each pericarp half was measured with a metal probe (0.3 mm diameter) driven into the sample at a speed of 1 mm min^−1^ while recording force and displacement. The pericarp puncture force (PPF; pericarp resistance) was determined to be the maximal force from the force–displacement curves. The values for each half were very similar and the mean for each segment was used. PPF values for 284–286 *R. raphanistrum* and 186–195 fruit segment halves were obtained. For measurements of intact fruit segments, segments were isolated and force was applied (top/bottom) on the outside of the fruit pod either on the MVR (36 segments) or perpendicular (34 segments) to the MVR.

Finite element (FE) simulation was conducted using Ansys 2016 (Ansys Inc., Canonsburg, USA). Cross-sections from *R. raphanistrum*, *R. pugioniformis* and *R. sativum* (for *R. sativum* from Vercellino *et al.* (2019)) were used to create a simplified vector graphic model of the fruit segments. The model was meshed with Mesh Modular (fine, high smoothing) and a force of 50 N was applied from the inside of the fruit segment and von Mises stress was calculated.

### Data analyses and statistics

Prism v10 (GraphPad Software, San Diego, CA, USA) was used to calculate mean ± standard error of the mean values for the figures, as well as for the statistical analyses of data. The data sets’ coefficient of variation (Fig. 5B) was calculated using GraphPad Prism v10 and is expressed as a percentage. This allows comparison of the scatter of variables and is calculated by dividing the standard deviation by the mean: coefficient of variation = (standard deviation/mean) × 100. It is the percentage of the expected deviation from the standard and is also known as ‘relative variability’.

**Fig. 1.**
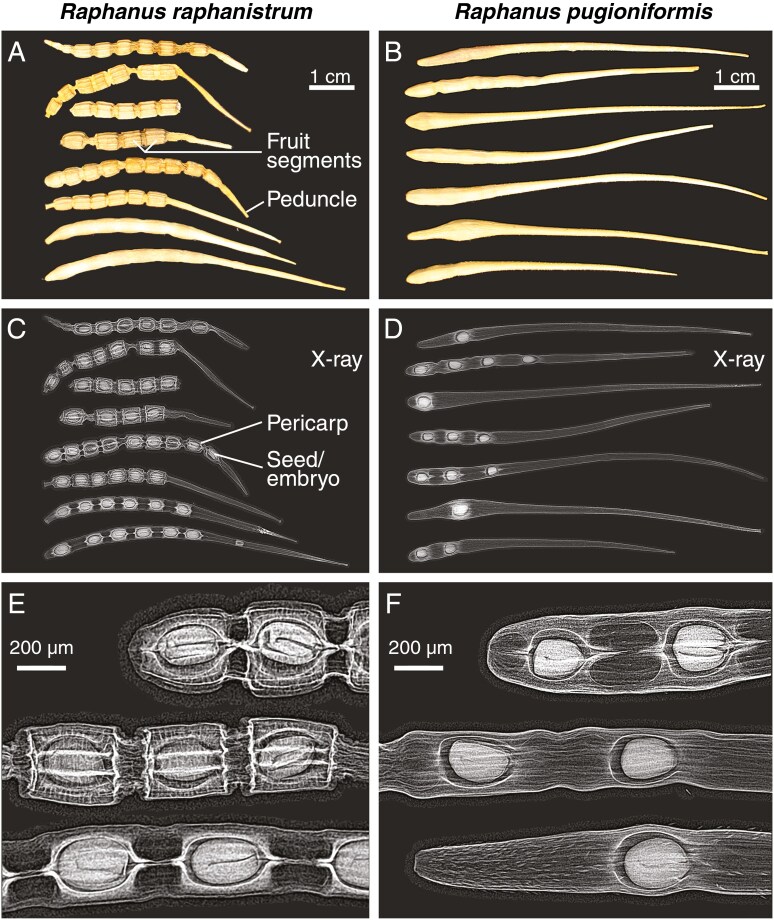
Fruit and seed morphology of wild radishes. (A) Mature fruits of *R. raphanistrum*; note that *R. raphanistrum* fruits may break apart into fruit segments. (B) Mature fruits of *R. pugioniformis*. (C) X-ray image of RR fruits shown in panel (A). (D) X-ray image of RP fruits shown in panel (B). (E) Enlargement of parts of panel (C) showing the internal morphology of *R. raphanistrum* fruit segments with seeds enclosed by pericarp (fruit wall) in seed chambers of individual fruit segments. (F) Enlargement of parts of panel (D) showing the internal morphology of *R. pugioniformis* fruits with seeds in seed chambers enclosed by pericarp (fruit wall). Note that the mature pericarp is dead tissue and that the X-ray imaging reveals distinct pericarp layers.

**Fig. 2.**
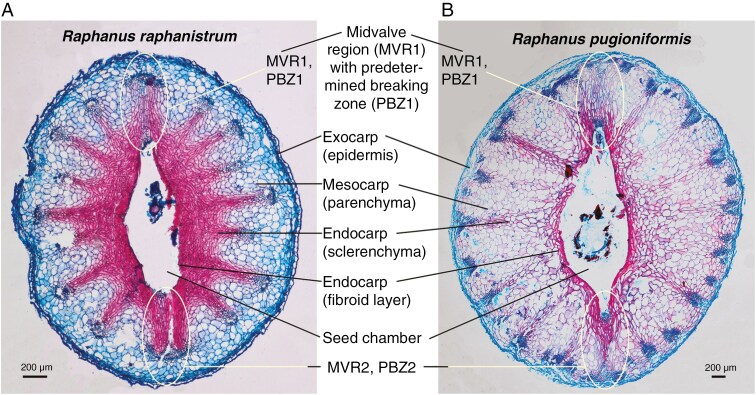
Microscopy of wild radish fruits with possible predetermined breaking zones (PDZs) in the midvalve regions (MVRs). (A) Safranin/astra blue-stained cross-section of an immature *R. raphanistrum* pericarp just prior to maturation drying. Lignified dead tissue stains red while living parenchyma tissue stains blue. Distinct layers of the pericarp and their cell types are indicated. (B) Safranin/astra blue-stained cross-section of an immature *Raphanus pugioniformis* pericarp.

**Fig. 3.**
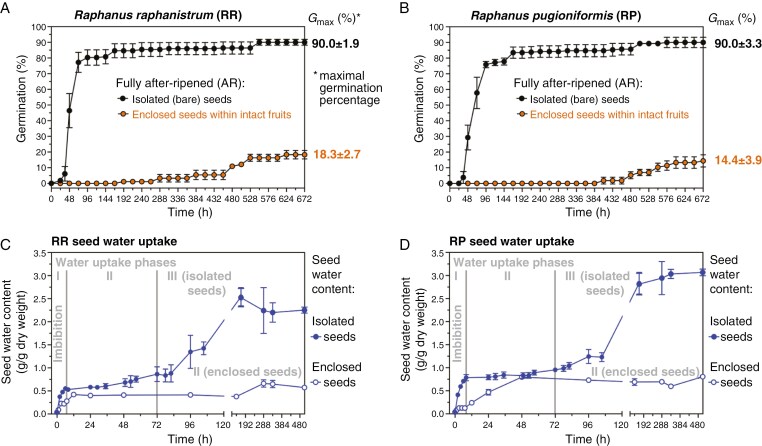
Effect of the pericarp on germination and water uptake of wild radish seeds. (A) Comparative germination analysis of fully after-ripened *R. raphanistrum* (RR) seeds enclosed by the pericarp within intact fruits with isolated (bare) seeds under standard germination conditions (25 °C 16 h light, 15 °C 8 h darkness). (B) Comparative germination analysis of fully after-ripened *R. pugioniformis* (RP) seeds enclosed by the pericarp within intact fruits and isolated (bare) seeds. Note that for RR and RP the pericarp-imposed constraint severely reduced the germination rates (speed) as well as the maximum germination percentages (*G*_max_). (C) Comparative analysis of water uptake into RR seeds. The three water uptake phases of isolated (bare) seeds during germination are indicated, with phase I (imbibition), phase II (metabolic activation) and phase III (radicle elongation and post-germination embryo growth) (Weitbrecht *et al.*, 2011). Imbibition and phase-II levels of water uptake were also evident for enclosed seeds inside fruits, but the transition to phase III was inhibited by the pericarp constraint. (D) Comparative analysis of water uptake into RP seeds. Mean values ± s.e.m. are presented for three to six (germination) and three (water uptake) replica plates each with 25 seeds or a total of at least six fruits.

**Fig. 4.**
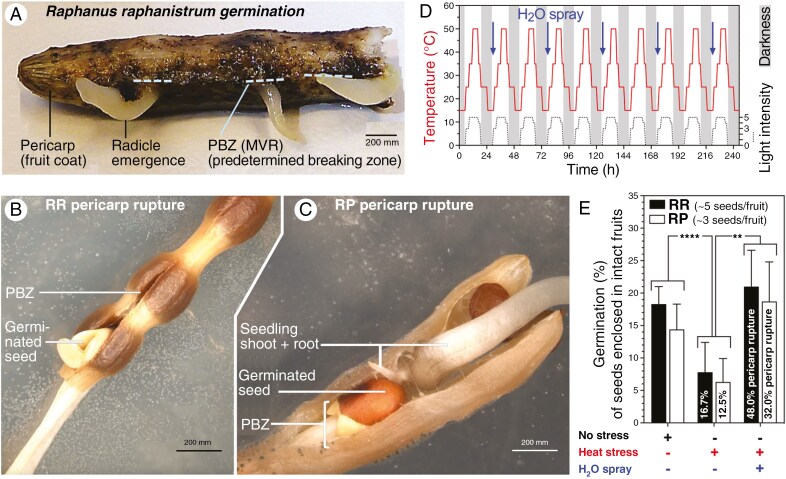
Effect of environmental factors on pericarp rupture and germination of wild radish seeds enclosed by the pericarp. (A) *R. raphanistrum* (RR) fruit with the pericarp (fruit wall) in the MVR ruptured at the PBZ and radicle emergence from germinated (enclosed) seeds within the fruit. (B) RR fruit pericarp rupture along the PBZ and germinated seed. (C) *R. pugioniformis* (RP) pericarp rupture along the PBZ, germinated seed and growing seedling. (D) Simulation program for extreme summer surface temperature conditions (heat stress) used for dry fruit incubation for up to 10 weeks; ‘H_2_O spray’ indicates morning dew simulation applied by spraying dry fruits every third day with a low amount of water. (E) Maximum germination percentages (*G*_max_) of RR and RP seeds enclosed in intact fruits under standard germination conditions (25 °C 16 h light, 15 °C 8 h darkness) without (left columns) and with (middle and right columns) preceding simulated heat stress treatment of dry fruits for up to 10 weeks (see panel D). The relative incidence of fruits with at least one germinated seed is also indicated. Note that the additional H_2_O spray promotes germination and reverts the negative effect of the heat stress. Mean values ± s.e.m. are presented for 24–26 fruits for each treatment containing on average five RR and three RP seeds per fruit.

## RESULTS

### *Morphological diversity of* Raphanus *dry indehiscent fruits*

Dry indehiscent fruits or fruit segments are the dispersal units of *Raphanus* species, including *R. sativus* (radish, an important root crop) (Vercellino *et al.*, 2019), its probable wild ancestor *R. raphanistrum* (wild radish, an invasive and noxious weed with global distribution) and *R. pugioniformis* (wild radish, a closely related endemic weed) (Cousens *et al.*, 2010; Ziffer-Berger *et al.*, 2020; Wasserstrom *et al.*, 2022). A comparison of *R. raphanistrum* and *R. pugioniformis* external and internal fruit morphology (Fig. 1) showed that the dispersed mature fruits of *R. raphanistrum* are more diverse and multi-segmented. Most have clearly visible constricted joints between their single-seeded segments of these lomentoid fruits. In the X-ray images (Fig. 1E) these joints appear to be composed of less dense material than the inner layers of the seed chambers. This is consistent with the observation that in the soil seed bank, *R. raphanistrum* fruits are broken into parts containing one or several fruit segments as dispersal units enclosing one or several seeds. In contrast to this, constricted joints and visible fruit segments are not or far less developed in *R. pugioniformis*, and all seeds are confined to the basal end of the fruits (Fig. 1). The entire indehiscent fruit is therefore the dispersal unit of *R. pugioniformis*. The two species differed in fruit size (Fig. 1), number of seeds per fruit (Wasserstrom *et al.*, 2022) and seed size (Supplementary Data Fig. S2). Seeds are enclosed by individual seed chambers of the pericarp (fruit coat, dead material in mature dry fruits) and the inner pericarp layers differed from the middle and outer pericarp layers in more dense material in the X-ray images (Fig. 1C–F).

Figure 2 demonstrates, using specific histostaining on fruits just prior to maturity, that the inner pericarp layers (endocarp) are fibroid and consist of cells with lignified secondary cell walls, whereas the mesocarp and exocarp are made up of living parenchyma cells. The tissues with lignified cells extend in a star-like pattern, especially in *R. raphanistrum*, towards the pericarp’s vascular bundles (Fig. 2A, B; Supplementary Data Fig. S3A). The seed chambers have the shape of ellipses with pointed ends located at the MVR. The unilocular seed chambers arise during fruit development from bilocular chambers separated by a septum (Supplementary Data Fig. S3B). In the mature fruit stage the remains of the membranous septum is retained and adheres to one side of the inner pericarp wall and seed surface (Supplementary Data Fig. S3C). This pattern was generated during fruit development by the thin and perforated septum being pushed towards either proximal side of the seed chamber. The unilocular fruits of *Raphanus* species are therefore only apparently septum-less.

Based on the fruit’s pericarp and seed chamber properties, the midvalve regions MVR1 and MVR2 could be predetermined breaking zones (PBZ1 and PBZ2) of the mature indehiscent fruits of *R. raphanistrum* and *R. pugioniformis* (Fig. 2). Seeds were positioned in the fruit’s seed chambers with the radicles adjacent to either MVR1 or MVR2 (Supplementary Data Fig. S4). There was no positioning preference for either MRV; roughly 1:1 MVR1/MVR2 ratios were obtained for the within-fruit radicle localization at the MVR1 or MVR2 position. Two PBZs corresponding to the two MVRs were also proposed for *R. sativus* fruits (Vercellino *et al.*, 2019), but in neither of these *Raphanus* species was their presence and functionality tested with biomechanical methods. The proposed pericarp-imposed mechanical dormancy and the mechanisms by which the enclosed seeds germinate are therefore based on assumptions and indirect evidence, and not on direct biomechanical analysis.

### *Ecophysiology of* Raphanus *germination and pericarp rupture*

Fully after-ripened isolated (bare) seeds of *R. raphanistrum* and *R. pugioniformis* were non-dormant and germinated readily with maximal germination percentages (*G*_max_) of 90 % under standard conditions (Fig. 3A, B). The completion of germination was followed by further embryo expansion and seedling growth (Supplementary Data Fig. S5a). In contrast to this, enclosed seeds within intact after-ripened fruits germinated much later, after 1 month, and only with 14–18 % *G*_max_ (Fig. 3A, B). No germination was observed for enclosed seeds within intact freshly harvested mature fruits during this period. Standard germination conditions (25 °C 16 h light, 15 °C 8 h darkness) were derived from analysis of weather data (Supplementary Data Fig. S1) of their native habitats, in which *R. raphanistrum* and *R. pugioniformis* germinate during late autumn. The weight of air-dried bare seeds of *R. raphanistrum* and *R. pugioniformis* differed (3.2 and 6.5 mg per seed, respectively), and seed sizes also differed considerably (Supplementary Data Fig. S2). *Raphanus pugioniformis* had an average of ~3 seeds per fruit, whereas *R. raphanistrum* had >5 seeds per fruit (Fig. 1C, D).

To test if the observed pericarp-mediated inhibition was caused by the presence of chemical compounds in the pericarp which act as inhibitors of seed germination, we compared unwashed (control) and washed (24 h in dH_2_O) after-ripened fruits. There was no statistical difference between the *G*_max_ values of unwashed and washed fruits (Supplementary Data Fig. S5B). We conclude that the pericarp-mediated inhibition is not caused by chemical inhibitors that leach from the pericarp. To test if the pericarp inhibits the germination of enclosed seeds by acting as a mechanical constraint to water uptake into the seed, we compared their water uptake patterns with bare seeds; water uptake into bare seeds typically has three phases (Weitbrecht *et al.*, 2011). There was no appreciable difference in the initial water uptake by imbibition (phase I) between enclosed and isolated (bare) seeds of *R. raphanistrum* (Fig. 3C). In contrast to this, the rate of water uptake by imbibition was ~6-fold slower in enclosed compared with bare seeds of *R. pugioniformis* (Fig. 3D). Similar seed water content characteristics for phase II (0.5–0.7 g H_2_O g^−1^ dry weight) were reached in enclosed and bare seeds for both species (Fig. 3C, D). Further water uptake at phase II/III, however, which is required for the completion of germination by radicle protrusion and subsequent embryo growth (phase III), was inhibited by the pericarp. The pericarp therefore acts as a mechanical constraint to phase-III water uptake, which keeps the seeds in phase II below the threshold for embryo growth and thereby severely delays or prevents the germination of enclosed seeds within intact after-ripened fruits.

The germination of enclosed seeds within intact fruits was associated with pericarp rupture along the MVR to permit radicle emergence from the fruit and subsequent seedling growth (Fig. 4A–C). Fruits detach from the mother plant by abscission during summer and persist during the hot and dry summer months (June to August) with low rainfall (<25 mm per month) and high air temperatures between 30 and 35 °C (Supplementary Data Fig. S1). Once dispersed into the seed bank, indehiscent fruits or fruit segments may experience high surface temperatures with >45 °C as possible extreme temperatures. To simulate extreme heat stress we exposed dry fruits to a day/night programme with fluctuating temperatures (Fig. 4D) for up to 10 weeks. Subsequent incubation under standard germination conditions demonstrated that the dry heat stress treatment reduced the maximum germination percentages (*G*_max_) of seeds enclosed in intact fruits 2.3-fold for both species (Fig. 4E). When, in addition to the heat stress, morning dew was simulated by spraying water every third day, this reduction was reversed to or above the *G*_max_ values of the untreated control. In addition, a higher percentage of fruits had at least one enclosed seed germinate (Fig. 4E).

### *Biomechanics of* Raphanus *germination and pericarp rupture*

**Fig. 5.**
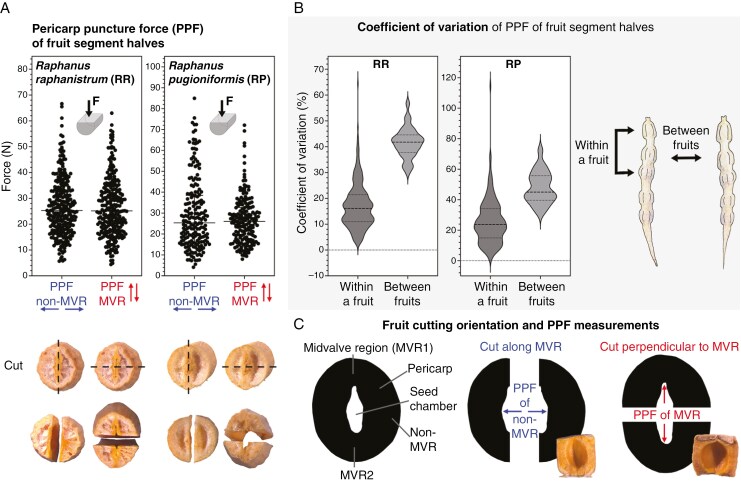
Biomechanical properties of fruit halves of *R. raphanistrum* (RR) and *R. pugioniformis* (RP). (A) Pericarp puncture force for fruit halves of RR (*n* = 286 and 284) and RP (*n* = 195 and 186) either cut open along the MVR or perpendicular to the MVR. No significant difference is found in either species, but both species show a large variation in observed forces. (B) The coefficient of variation (relative variability) is higher when comparing different fruits with each other compared with individual fruit pods within one fruit for RR and RP. (C) Fruit segment cutting orientations used in the pericarp puncture force experiments. Fruits were either cut along the MVR region and probed at the non-MVR zone or cut perpendicular to the MVR and then probed at the MVR zone.

To investigate if the MVRs, which are located at the pointed ends of the seed chambers (Fig. 2) and are where the pericarp splits open to permit radicle emergence (Fig. 4), we conducted direct biomechanical analysis (Fig. 5). Mature fruits were cut either along or perpendicular to the MVR1 and MVR2 plane (Fig. 5C) and the PPF was quantified by applying force from the inside on a fruit segment half. Figure 5A demonstrates that *R. raphanistrum* and *R. pugioniformis* PPF at the MVR and the non-MVR pericarp regions did not differ significantly and was highly variable. This was from 5.7 to 66.7 N along the MVR and from 4.4 to 63 N perpendicular to the MVR for *R. raphanistrum*, and from 4.1 to 85 N along the MVR and from 7.9 to 72.1 N perpendicular to the MVR for *R. pugioniformis*. The mechanical properties of the fruit segment halves measured at the MVR and non-MVR region did therefore not differ. PPF values for breaking the pericarps of *R. raphanistrum* compared with *R. pugioniformis* were not significantly different, although the maximum achieved forces in *R. pugioniformis* were higher (Fig. 5A). Both species showed a very high variability in the breaking forces (PPF) (Fig. 5B). The coefficient of variation (relative variability), which equals the standard deviation divided by the mean, was higher when comparing different fruits with each other in comparison to comparing individual fruit segments within one fruit. *Raphanus raphanistrum* and *R. pugioniformis* both therefore produce whole fruits with either relatively low or relatively high PPF.

**Fig. 6.**
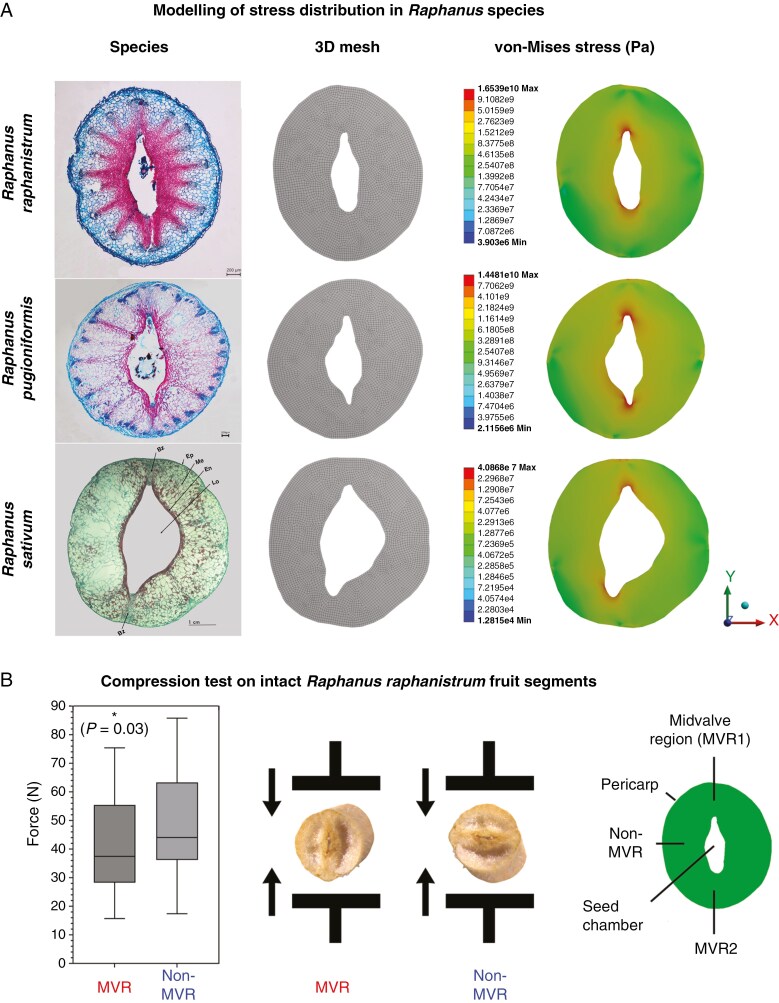
Biomechanics and stress distribution in intact fruit segments. (A) Finite element model of fruit segments of *R. raphanistrum*, *R. pugioniformis* and *R. sativum* based on their cross-sections; the *R. sativum* microscopy image (Vercellino *et al.*, 2019) is reprinted with permission. The 3D mesh of each fruit segment was loaded with a force of 50 N, applied from the inside onto the pericarp, and the von Mises stress in pascals is depicted. Von-Mises stress shows stress peaks in the MVR for each of the tested species. (B) Box plots with Tukey whiskers and medians for the pericarp breaking force in a compression test either along the MVR (*n* = 36) or perpendicular to the MVR (*n* = 34) for *R. raphanistrum*. Force applied in line with the MVR is significantly lower (*P* = 0.03).

Finite element simulation is a powerful computational tool in fracture mechanics to analyse the influence of shape/geometry on the stress and strain distribution in materials (Hernandez and Belles, 2007; Li *et al.*, 2018, 2023; Sachse *et al.*, 2020). The FE model revealed that for all tested *Raphanus* species (*R. raphanistrum*, *R. pugioniformis*, *R. sativum*) the stresses obtained within fruit segments, when force is applied from the inside mimicking a germinating seed, are highest at the MVR (Fig. 6A). This suggests that this is the region where *Raphanus* fruits are most likely to open, which aligns with the observation of pericarp rupture during indehiscent fruit germination, and supports the hypothesis that the MVRs contain a PBZ.

To investigate the importance of the shape as a morphological property, compression tests were performed on intact fruit segments of *R. raphanistrum* to analyse the biomechanics of the entire (uncut) fruit structure. Applying compression force in line with the MVR of the fruit segments revealed a significantly lower median breaking force of 37.5 N compared with 44.1 N perpendicular to the MVR (Fig. 6B). No matter the orientation of the fruit segment, the majority of pericarps failed along the MVR. Taking these results together (Fig. 6), we conclude that the shape of the MVR pericarp defines the PBZ of *Raphanus* indehiscent fruits.

## DISCUSSION

### *Roles of pericarp-imposed mechanical dormancy in* Raphanus *seed germination*

The dispersal units of *Raphanus* species are dry mature multiseeded indehiscent siliques or their disaggregated fruit segments (*R. raphanistrum*) in which the seeds are enclosed by a water-permeable rigid pericarp (Hoffmann, 1872; Mekenian and Willemsen, 1975; Cousens *et al.*, 2010; Heredia and Ellstrand, 2014; Tricault *et al.*, 2017; Vercellino *et al.*, 2019; Kebaso *et al.*, 2020; Ziffer-Berger *et al.*, 2020; Wasserstrom *et al.*, 2022). The pericarp plays an important role in environmental adaptation by imposing dormancy in radish (this work and Cousens *et al.*, 2010; Tricault *et al.*, 2017; Vercellino *et al.*, 2019) and many other Brassicaceae species with indehiscent fruits (e.g. Lu *et al.*, 2015, 2017*a*, *b*; Zhou *et al.*, 2015; Sperber *et al.*, 2017; Mohammed *et al.*, 2019; Chandler *et al.*, 2024). Bare seeds of *R. raphanistrum*, *R. pugioniformis* and *R. sativus* isolated from after-ripened fruits (after prolonged dry storage) were non-dormant and germinated readily within ~2 d with a *G*_max_ of ~90 % (this work and Cousens *et al.*, 2010; Vercellino *et al.*, 2019). In contrast to this, germination of enclosed seeds within after-ripened fruits of these species required ~2–6 weeks and only reached a *G*_max_ of 15–35 %. Examples for pericarp-imposed chemical dormancy by leaching germination-inhibiting compounds such as abscisic acid include *Lepidium draba*, *Anastatica hierochuntica* and *Beta vulgaris* (Hermann *et al.*, 2007; Ignatz *et al.*, 2019; Mohammed *et al.*, 2019; Khadka *et al.*, 2020). There was no evidence for pericarp-derived chemical dormancy for *R. raphanistrum*, *R. pugioniformis* (this work) and *R. sativus* (Vercellino *et al.*, 2019).

The results from water uptake experiments comparing bare and enclosed seeds of these *Raphanus* species provide strong evidence for pericarp-imposed mechanical dormancy by decreasing the imbibition rates and limiting final seed water contents (this work and Cousens *et al.*, 2010; Vercellino *et al.*, 2019). The pericarp therefore effectively limited the transition from water uptake phase II to phase III, which is required for embryo expansion to complete seed germination. Pericarp-imposed mechanical dormancy of *Lepidium didymum* and *Tilia miqueliana* was released by microbial activity biomechanically weakening the hard fruit coat to permit germination (Sperber *et al.*, 2017; Wu *et al.*, 2023). While there is no evidence for the involvement of microbial activity in *Raphanus* fruits, there is evidence for roles of the pericarp-imposed mechanical constraint to seed water uptake in soil seed bank persistence. Burial experiments demonstrated that pericarp thickness of *R. raphanistrum* populations was positively associated with seed longevity in the soil seed bank (Tricault *et al.*, 2017). The water-absorbing capacity of the pericarp may provide an internal environment that keeps the seed of feral *R. sativus* moist after a period of moderate rainfall (Vercellino *et al.*, 2019). Wetting/drying cycles combined with limiting the final seed water contents and appropriate temperatures may provide a seed priming effect, release primary seed dormancy, or induce secondary dormancy in unpredictable environments (Cousens *et al.*, 2010; Tricault *et al.*, 2017; Vercellino *et al.*, 2019). *Raphanus sativus* seeds are very heat-resistant, and their viability remained high at >50 °C even at high seed moisture content (Waggoner, 1917). In agreement with this and a protective role of the pericarp, we found that *R. raphanistrum* and *R. pugioniformis* seeds remained viable even during prolonged incubation of dry fruits in conditions mimicking alternating soil seed bank temperatures (up to 50 °C) and morning dew.

### *Pericarp morphology and development of* Raphanus *indehiscent unilocular siliques*

Fruit development generally begins with fertilization, when the carpel wall starts to differentiate into the three pericarp layers (endocarp, mesocarp, exocarp) (Roeder and Yanofsky, 2006; Dardick and Callahan, 2014; Pabon-Mora *et al.*, 2014; Eldridge *et al.*, 2016; Bobrov and Romanov, 2019). Brassicaceae fruits originate from two fused carpels that develop into bilocular siliques or silicles composed of two valves separated by a septum that spans the valve margins. Our microscopic analysis of *Raphanus* fruit development (Supplementary Data Fig. S3) revealed that in the early stages a septum spans between the valve margins, which is consistent with the bilocular origin of the apparently unilocular mature siliques. A membranous septum is retained and adheres to one side of the inner pericarp wall and seed surface in mature siliques. This structure is generated during fruit development by the thin and perforated septum being pushed towards either proximal side of the seed chamber. The indehiscent fruits of *Raphanus* species therefore are apparently septum-less with the almost invisible septum being pulled through the fruit in a wavelike manner (Supplementary Data Fig. S3 and Hoffmann, 1872; Hannig, 1901; Brückner, 2000). The genetic mechanisms regulating the development of dry dehiscent fruits have been identified in *Arabidopsis thaliana* and found to be fairly conserved across the Brassicaceae and less conserved in other core eudicots (Roeder and Yanofsky, 2006; Avino *et al.*, 2012; Mühlhausen *et al.*, 2013; Dardick and Callahan, 2014; Eldridge *et al.*, 2016). The evolutionary transition from dehiscent to indehiscent Brassicaceae fruits was caused by a change in the control of valve margin identity genes (Avino *et al.*, 2012; Mühlhausen *et al.*, 2013; Eldridge *et al.*, 2016). Very little is known about the morphological patterns and germination mechanisms in species with indehiscent fruits.

We found that pericarp rupture and radicle emergence of imbibed unilocular *Raphanus* fruits occurs in the MVR and not at the valve margin. Using specific histostaining on fruits just prior to maturity (Fig. 2), we demonstrated that the endocarp contains lignified secondary cell walls, whereas the mesocarp and exocarp are made up of living parenchyma cells. A region with reduced lignification was identified in the hard endocarp of *Lepidium didymum* fruit valves to function as the crack initiation zone from where a PBZ guides its propagation in an ‘opening fracture mode’ along the MVR (Sperber *et al.*, 2017). This crack initiation zone was localized in the distal MVR adjacent to the seed’s radicle end, which was always oriented towards the distal MVR. In contrast to this, in *Raphanus* fruits the seed’s radicle end was localized adjacent to either MVR1 or MVR2 with equal distribution and no preference for either MVR (Supplementary Data Fig. S4). Also, no single specific region with reduced lignification was obvious (Fig. 2). Fruit shape diversity in the Brassicaceae is generated by varying patterns of anisotropy (Eldridge *et al.*, 2016). The different morphological shapes of Brassicaceae siliques are generated by spatially distinct growth in the late phase of fruit development. Modelling of *A. thaliana* fruit growth showed that fruits elongate with a cylindrical shape and near-circular circumference, while in *Capsella bursa-pastoris* and *Lepidium* spp. laterally flattened spheroid-shaped fruits with oval circumferences are produced. An important point is that the diversity in Brassicaceae fruit shapes depends on spatially distinct growth in the valve margin region, for which expression patterns of the valve margin pathway genes are known (Avino *et al.*, 2012; Mühlhausen *et al.*, 2013; Eldridge *et al.*, 2016), as well as in the MVR1/MVR2, for which the developmental factors are unknown. Spatially differential growth of the pericarp during *Raphanus* fruit development therefore results in seed chambers that have the shape of ellipses with pointed ends located at the MVRs.

### *Biomechanics of* Raphanus *pericarp rupture and morphological fruit variability*

Direct biomechanical analysis of *Raphanus* pericarp rupture by applying force to the inside of cut fruit segment halves revealed that their PPF did not differ between the MVR and non-MVR (Fig. 5). We concluded from this, combined with the finding that pericarp rupture occurred only along the MVR, that the shape of the intact fruit segments must provide the spatial information for the location of the PBZ. Modelling of the stress distribution in *Raphanus* fruit segments indeed confirmed that the pointed ends of the elliptic seed chambers provide PBZ1 and PBZ2 which are located in MVR1 and MVR2, respectively (Fig. 6). This finding was experimentally confirmed with a compression test on intact fruit segments, which demonstrated that a significantly lower force was required for the MVR as compared with the non-MVR. To our knowledge such a comprehensive biomechanical analysis (cut versus intact fruit segments, FE modelling and direct force measurement) has not been conducted for the pericarp of any other species. Finite element modelling combined with direct force measurements were conducted for tomato as a soft fruit (Li *et al.*, 2023) and for sunflower dry fruits as a biomechanical approach to improve hullability (Hernandez and Belles, 2007). Our multiscale biomechanics approach identified the PBZ in *Raphanus* siliques based on the pericarp shape of the MVR.

Further to this, our direct biomechanical analysis also revealed great variability in the within-fruit and between-fruits breaking forces (PPF). *Raphanus raphanistrum* and *R. pugioniformis* therefore produce siliques with either relatively low or relatively high PPF. We propose that this high variability in biomechanical fruit properties is a key component of their soil seed bank persistence. It contributes to the high phenotypic variability that enables *Raphanus* species to adapt and thrive in different environments (Tricault *et al.*, 2017; Kebaso *et al.*, 2020; Ziffer-Berger *et al.*, 2020; Bhattacharya *et al.*, 2022; Wasserstrom *et al.*, 2022). *Raphanus raphanistrum* seeds are generally viable for 6 years, but can remain viable for up to 20 years if buried deep in the soil. Fruit properties and environmental conditions likely to cause pericarp degradation of *Raphanus* species enhance the exit from the soil seed bank by germination and seedling emergence (Cousens *et al.*, 2010; Tricault *et al.*, 2017; Vercellino *et al.*, 2019). Variable pericarp properties of indehiscent fruits contribute to seed longevity and dormancy and thereby spread seed persistence in space and time. This includes variability in pericarp thickness (Eslami *et al.*, 2010; Tricault *et al.*, 2017) and/or mechanical strength (this work and Heredia and Ellstrand, 2014). In other Brassicaceae with indehiscent fruits the pericarp is known to contribute to soil seed bank persistence, to retain seed viability over repetitive drying/wetting cycles, and to ensure that seeds germinate during the cool season for seedling survival in desert environments (Neya *et al.*, 2008; Lu *et al.*, 2015, 2017*a*, *b*; Zhou *et al.*, 2015). Variability in pericarp-imposed mechanical dormancy therefore provides a maternally controlled bet-hedging strategy to spread germination over an extended period of time.

## Conclusions

Our comparative study of the dry indehiscent fruits of *Raphanus* species demonstrated that their hard pericarp imposes mechanical dormancy by preventing full phase-II water uptake of the enclosed seeds. Even in fully after-ripened siliques this delayed germination by >2 months and reduced the maximal seed germination percentage to <20 %. Biomechanics and imaging (microscopy and X-ray) analysis of developing and mature *Raphanus* fruits showed that the apparently unilocular fruits develop from two valves fused together at the valve margins with the remains of the septum visible in mature siliques, and that pericarp rupture to aid radicle emergence during seed germination occurs along a PBZ identified in each of the MVRs. Our multiscale biomechanics approach revealed that the internal shape of seed chambers in the indehiscent siliques of *R. raphanistrum* (wild radish, global weed), *R. pugioniformis* (wild radish, endemic weed) and *R. sativus* (feral radish derived from cultivated biotype) is a key feature of the PBZ/MVR. We conclude that the Brassicaceae, with their morphological diversity in fruit shapes generated by varying patterns of anisotropic growth, provide excellent systems to comparatively investigate the biomechanical, molecular and ecological mechanisms by which dehiscent and indehiscent fruits and seeds contribute to environmental adaptation. We conclude that the high variability in pericarp-imposed dormancy of *Raphanus* weed indehiscent fruits provides a bet-hedging strategy that defines their soil seed bank persistence (wetting/drying and warm/cold cycles) and enables the spread of germination across time and seasons.

## SUPPLEMENTARY DATA

Supplementary data are available at *Annals of Botany* online and consist of the following. Figure S1: seasonal weather and simulated weather of the wild radishes’ native habitats. Figure S2: morphological properties of *Raphanus raphanistrum* (RR) and *Raphanus pugioniformis* (RP) seeds. Figure S3. *Raphanus raphanistrum* (RR) fruit development and identification of the remains of the septum in mature *Raphanus* spp. fruits. Figure S4. frequency distribution of seed positioning within the seed chambers with the radicles adjacent to either midvalve region MVR1 or MVR2. Figure S5. Germination and seedling growth of *Raphanus raphanistrum* (RR) and *Raphanus pugioniformis* (RP).

mcaf015_suppl_Supplementary_Materials

## Data Availability

The data generated in this study are available online in the electronic supplementary material and through ﬁgshare: https://doi.org/10.17637/rh.27275832.v1.

## References

[CIT0001] Arshad W , Sperber K, Steinbrecher T, et al 2019. Dispersal biophysics and adaptive significance of dimorphic diaspores in the annual *Aethionema arabicum* (Brassicaceae). The New Phytologist 221: 1434–1446. 30230555 10.1111/nph.15490 PMC6492137

[CIT0002] Avino M , Kramer EM, Donohue K, Hammel AJ, Hall JC. 2012. Understanding the basis of a novel fruit type in Brassicaceae: conservation and deviation in expression patterns of six genes. EvoDevo 3: 20. 22943452 10.1186/2041-9139-3-20 PMC3503883

[CIT0003] Baskin CC , Baskin JM. 2014. Seeds, ecology, biogeography, and evolution of dormancy and germination. San Diego: Academic Press.

[CIT0004] Batlla D , Ghersa CM, Benech-Arnold RL. 2020. Dormancy, a critical trait for weed success in crop production systems. Pest Management Science 76: 1189–1194. 31800163 10.1002/ps.5707

[CIT0005] Bhattacharya S , Grone F, Przesdzink F, et al 2022. ‘Root of all success’: plasticity in root architecture of invasive wild radish for adaptive benefit. Frontiers in Plant Science 13: 1035089. 36466265 10.3389/fpls.2022.1035089 PMC9709435

[CIT0006] Bobrov AVFC , Romanov MS. 2019. Morphogenesis of fruits and types of fruit of angiosperms. Botany Letters 166: 366–399.

[CIT0007] Brückner C. 2000. Clarification of the carpel number in Papaverales, Caparales, and Berberidaceae. The Botanical Review 66: 155–307.

[CIT0008] Chandler JO , Wilhelmsson PKI, Fernandez-Pozo N, et al 2024. The dimorphic diaspore model *Aethionema arabicum* (Brassicaceae): distinct molecular and morphological control of responses to parental and germination temperatures. Plant Cell 36: 2465–2490. 38513609 10.1093/plcell/koae085 PMC11218780

[CIT0009] Collinson ME , Manchester SR, Wilde V. 2012. Fossil fruits and seeds of the Middle Eocene Messel biota, Germany. Abhandlungen der Senckenberg Gesellschaft für Naturforschung 570: 1–249.

[CIT0010] Cousens RD , Young KR, Tadayyon A. 2010. The role of the persistent fruit wall in seed water regulation in *Raphanus raphanistrum* (Brassicaceae). Annals of Botany 105: 101–108. 19889801 10.1093/aob/mcp268 PMC2794069

[CIT0011] Dardick C , Callahan AM. 2014. Evolution of the fruit endocarp: molecular mechanisms underlying adaptations in seed protection and dispersal strategies. Frontiers in Plant Science 5: 284. 25009543 10.3389/fpls.2014.00284 PMC4070412

[CIT0012] Eldridge T , Langowski L, Stacey N, et al 2016. Fruit shape diversity in the Brassicaceae is generated by varying patterns of anisotropy. Development 143: 3394–3406. 27624834 10.1242/dev.135327 PMC5047655

[CIT0013] Eslami SV , Gill GS, McDonald G. 2010. Effect of water stress during seed development on morphometric characteristics and dormancy of wild radish (*Raphanus raphanistrum* L.) seeds. International Journal of Plant Production 4: 159–168.

[CIT0014] Ezra Z , Levavi L, Bar-B. 2023. The load-bearing mechanism of plant wings: a multiscale structural and mechanical analysis of the *T. tipu* samara. Acta Biomaterialia 158: 423–434. 36563776 10.1016/j.actbio.2022.12.040

[CIT0015] Finch-Savage WE , Leubner-Metzger G. 2006. Seed dormancy and the control of germination. The New Phytologist 171: 501–523. 16866955 10.1111/j.1469-8137.2006.01787.x

[CIT0016] Grafi G. 2020. Dead but not dead end: multifunctional role of dead organs enclosing embryos in seed biology. International Journal of Molecular Sciences 21: 8024. 33126660 10.3390/ijms21218024 PMC7662896

[CIT0017] Hannig E. 1901. Untersuchungen über die Scheidewände der Cruciferenfrüchte. Botanische Zeitung 59: 207–245.

[CIT0018] Heredia SM , Ellstrand NC. 2014. Novel seed protection in the recently evolved invasive, California wild radish, a hybrid *Raphanus* sp. (Brassicaceae). American Journal of Botany 101: 2043–2051. 25480701 10.3732/ajb.1400036

[CIT0019] Hermann K , Meinhard J, Dobrev P, et al 2007. 1-Aminocyclopropane-1-carboxylic acid and abscisic acid during the germination of sugar beet (*Beta vulgaris* L.): a comparative study of fruits and seeds. Journal of Experimental Botany 58: 3047–3060. 17761730 10.1093/jxb/erm162

[CIT0020] Hernandez LF , Belles PM. 2007. A 3-D finite element analysis of the sunflower (*Helianthus annuus* L.) fruit. Biomechanical approach for the improvement of its hullability. Journal of Food Engineering 78: 861–869.

[CIT0021] Hill AW. 1933. The method of germination of seeds enclosed in a stony endocarp. Annals of Botany 47: 873–887.

[CIT0022] Hoffmann H. 1872. Ueber Raphanus-Früchte. Botanische Zeitung 30: 482–487.

[CIT0023] Hourston JE , Steinbrecher T, Chandler JO, et al 2022. Cold-induced secondary dormancy and its regulatory mechanisms in *Beta vulgaris*. Plant, Cell & Environment 45: 1315–1332. 10.1111/pce.14264 PMC9305896 35064681

[CIT0024] Ignatz M , Hourston JE, Tureckova V, et al 2019. The biochemistry underpinning industrial seed technology and mechanical processing of sugar beet. Planta 250: 1717–1729. 31414204 10.1007/s00425-019-03257-5 PMC6790189

[CIT0025] Kebaso L , Frimpong D, Iqbal N, et al 2020. Biology, ecology and management of *Raphanus raphanistrum* L.: a noxious agricultural and environmental weed. Environmental Science and Pollution Research International 27: 17692–17705. 32246421 10.1007/s11356-020-08334-x

[CIT0026] Khadka J , Raviv B, Swetha B, et al 2020. Maternal environment alters dead pericarp biochemical properties of the desert annual plant *Anastatica hierochuntica* L. PLoS One 15: e0237045. 32735576 10.1371/journal.pone.0237045 PMC7394380

[CIT0027] Lazarus BS , Leung V, Luu RK, et al 2023. Jackfruit: composition, structure, and progressive collapsibility in the largest fruit on the Earth for impact resistance. Acta Biomaterialia 166: 430–446. 37121367 10.1016/j.actbio.2023.04.040

[CIT0029] Li H , Li JS, Yuan H. 2018. A review of the extended finite element method on macrocrack and microcrack growth simulations. Theoretical and Applied Fracture Mechanics 97: 236–249.

[CIT0028] Li DD , Liu Y, Fadiji T, Li ZG, Okasha M. 2023. Analysis of the correlation between mesocarp biomechanics and its cell turgor pressure: a combined FEM-DEM investigation for irrigation-caused tomato cracking. Journal of Texture Studies 54: 206–221. 36116087 10.1111/jtxs.12720

[CIT0032] Lu JJ , Zhou YM, Tan DY, Baskin CC, Baskin JM. 2015. Seed dormancy in six cold desert Brassicaceae species with indehiscent fruits. Seed Science Research 25: 276–285.

[CIT0030] Lu JJ , Tan DY, Baskin CC, Baskin JM. 2017a. Delayed dehiscence of the pericarp: role in germination and retention of viability of seeds of two cold desert annual Brassicaceae species. Plant Biology (Stuttgart, Germany) 19: 14–22. 27037632 10.1111/plb.12457

[CIT0031] Lu JJ , Tan DY, Baskin CC, Baskin JM. 2017b. Role of indehiscent pericarp in formation of soil seed bank in five cold desert Brassicaceae species. Plant Ecology 218: 1187–1200.

[CIT0033] Mekenian MR , Willemsen RW. 1975. Germination characteristics of *Raphanus raphanistrum*. Bulletin of the Torrey Botanical Club 102: 243–252.

[CIT0034] Mohammed S , Turckova V, Tarkowska D, Strnad M, Mummenhoff K, Leubner-Metzger G. 2019. Pericarp-mediated chemical dormancy controls the fruit germination of the invasive hoary cress (*Lepidium draba*), but not of hairy whitetop (*Lepidium appelianum*). Weed Science 67: 560–571.

[CIT0035] Mühlhausen A , Lenser T, Mummenhoff K, Theissen G. 2013. Evidence that an evolutionary transition from dehiscent to indehiscent fruits in *Lepidium* (Brassicaceae) was caused by a change in the control of valve margin identity genes. The Plant Journal 73: 824–835. 23173897 10.1111/tpj.12079

[CIT0036] Mummenhoff K , Polster A, Muhlhausen A, Theissen G. 2009. *Lepidium* as a model system for studying the evolution of fruit development in Brassicaceae. Journal of Experimental Botany 60: 1503–1513. 19052256 10.1093/jxb/ern304

[CIT0037] Nakabayashi K , Leubner-Metzger G. 2021. Seed dormancy and weed emergence: from simulating environmental change to understanding trait plasticity, adaptive evolution, and population fitness. Journal of Experimental Botany 72: 4181–4185. 34048571 10.1093/jxb/erab150 PMC8163051

[CIT0038] Neya O , Hoekstra FA, Golovina EA. 2008. Mechanism of endocarp-imposed constraints of germination of *Lannea microcarpa* seeds. Seed Science Research 18: 13–24.

[CIT0039] Pabon-Mora N , Wong GKS, Ambrose BA. 2014. Evolution of fruit development genes in flowering plants. Frontiers in Plant Science 5: ARTN 300. 10.3389/fpls.2014.00300 PMC4071287 25018763

[CIT0040] Roeder AH , Yanofsky MF. 2006. Fruit development in *Arabidopsis*. The Arabidopsis book 4: e0075. 22303227 10.1199/tab.0075 PMC3243326

[CIT0041] Sachse R , Westermeier A, Mylo M, et al 2020. Snapping mechanics of the Venus flytrap (*Dionaea muscipula*). Proceedings of the National Academy of Sciences of the United States of America 117: 16035–16042. 32571929 10.1073/pnas.2002707117 PMC7355038

[CIT0042] Sperber K , Steinbrecher T, Graeber K, et al 2017. Fruit fracture biomechanics and the release of *Lepidium didymum* pericarp-imposed mechanical dormancy by fungi. Nature Communications 8: 1868. 10.1038/s41467-017-02051-9 PMC5709442 29192192

[CIT0043] Steinbrecher T , Leubner-Metzger G. 2017. The biomechanics of seed germination. Journal of Experimental Botany 68: 765–783. 27927995 10.1093/jxb/erw428

[CIT0044] Steinbrecher T , Leubner-Metzger G. 2018. Tissue and cellular mechanics of seeds. Current Opinion in Genetics and Development 51: 1–10. 29571069 10.1016/j.gde.2018.03.001

[CIT0045] Tricault Y , Matejicek A, Darmency H. 2017. Variation of seed dormancy and longevity in *Raphanus raphanistrum* L. Seed Science Research 28: 34–40.

[CIT0047] Vercellino RB , Pandolfo CE, Cerrota A, Cantamutto M, Presotto A. 2019. The roles of light and pericarp on seed dormancy and germination in feral *Raphanus sativus* (Brassicaceae). Weed Research 59: 396–406.

[CIT0046] Vercellino RB , Hernandez F, Presotto A. 2023. The role of intraspecific crop-weed hybridization in the evolution of weediness and invasiveness: cultivated and weedy radish (*Raphanus sativus*) as a case study. American Journal of Botany 110: e16217. 37659092 10.1002/ajb2.16217

[CIT0048] Waggoner HD. 1917. The viability of radish seeds (*Raphanus sativus* L.) as affected by high temperatures and water content. American Journal of Botany 4: 299–313.

[CIT0049] Wasserstrom H , Ziffer-Berger J, Barzilai M, Mummenhoff K, Barazani O. 2022. Phenotypic variation of wild radishes *Raphanus pugioniformis* and *R. raphanistrum* associated with local conditions in the southeast Mediterranean. Flora 287: 151997.

[CIT0050] Weitbrecht K , Müller K, Leubner-Metzger G. 2011. First off the mark: early seed germination. Journal of Experimental Botany 62: 3289–3309. 21430292 10.1093/jxb/err030

[CIT0051] Wu Y , Sun XR, Pritchard HW, Shen YB, Wu XQ, Peng CY. 2023. The metagenomics of soil bacteria and fungi and the release of mechanical dormancy in hard seeds. Frontiers in Plant Science 14: 1187614. 37441178 10.3389/fpls.2023.1187614 PMC10335401

[CIT0052] Zhou YM , Lu JJ, Tan DY, Baskin CC, Baskin JM. 2015. Seed germination ecology of the cold desert annual *Isatis violascens* (Brassicaceae): two levels of physiological dormancy and role of the pericarp. PLoS One 10: e0140983. 26513241 10.1371/journal.pone.0140983 PMC4626048

[CIT0053] Ziffer-Berger J , Waitz Y, Behar E, et al 2020. Seed dispersal of wild radishes and its association with within-population spatial distribution. BMC Ecology 20: 30. 32393235 10.1186/s12898-020-00297-4 PMC7212605

