# Supplementary material for: Comparative pericarp biomechanics and germination physiology of Raphanus raphanistrum and Raphanus pugioniformis indehiscent fruits
Source: Ann Bot. 2025 Jan 27;135(5):977–90. doi: 10.1093/aob/mcaf015 (PMC12064425; doi:10.1093/aob/mcaf015)
Supplement: mcaf015_suppl_Supplementary_Materials [file mcaf015_suppl_supplementary_materials.docx]

**Comparative pericarp biomechanics and germination physiology of *Raphanus raphanistrum* and *Raphanus pugioniformis* indehicent fruits**

**Tina Steinbrecher[1], Samik Bhattacharya[2,3], Jonathan Binder[1], Katharina Kleemeier[2], Felix Przesdzink[2], Franziska Groene[2,4], Kyra Jacoblinnert[2], Klaus Mummenhoff[2,4,*], Gerhard Leubner-Metzger[1,5,*]**

[1] Seed Biology and Technology Group, Department of Biological Sciences, Royal Holloway University of London, TW20 0EX, Egham, United Kingdom
[2] Department of Biology, Botany, University of Osnabrück, Osnabrück, Germany
[3] Resolve Biosciences, Alfred-Nobel-Straße 10, 40789 Monheim am Rhein, Germany
[4] seedalive, Albert-Einstein-Strasse 30, 49076 Osnabrück, Germany
[5] Laboratory of Growth Regulators, Faculty of Science, Palacký University and Institute of Experimental Botany, Czech Academy of Sciences, CZ-78371 Olomouc, Czech Republic

**Journal:** Annals of Botany

**Supplementary Data**

https://doi.org/10.1093/aob/mcaf015

© 2025 The Authors. Published by Oxford University Press on behalf of the Annals of Botany Company. This is an open access article distributed under the terms of the Createve Commons Attribution License (https://creativecommons.org/licenses/by/4.0/) which permits unrestricted reuse, distribution, and reproduction in any medium, provided the original work is properly cited.

## A *Raphanus* seed collection sites in their native habitats in Israel

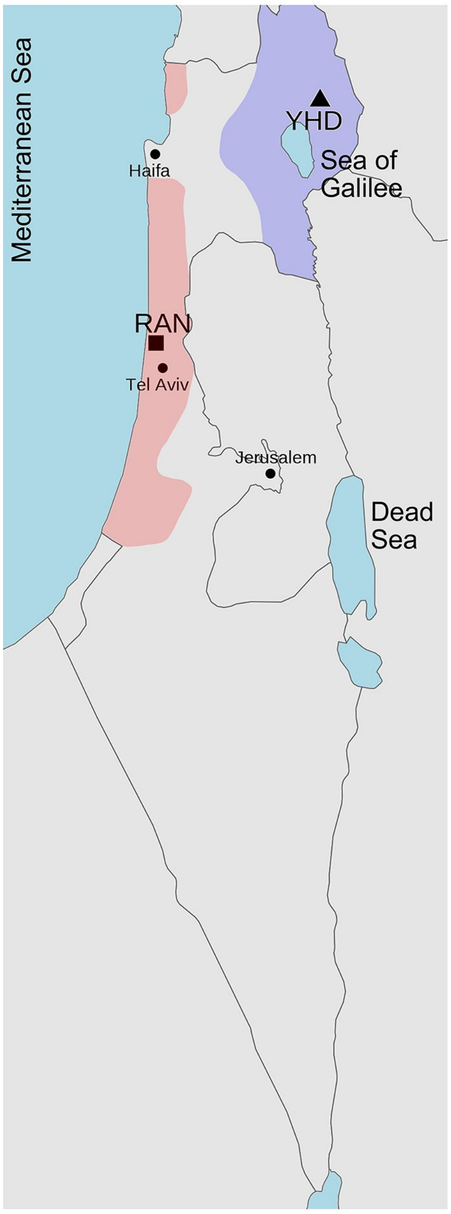


## D Simulation of future weather in *Raphanus* experiments

**Summer (June-August) simulation of typical air temperatures, relative light intensities**

| Time | 00:00 – 6:00 | 6:00 – 9:00 | 9:00 - 12:00 | 12:00 – 17:00 | 17:00 - 19:00 | 19:00 - 24:00 |
|---|---|---|---|---|---|---|
| Temperature | 20 | 25 | 30 | 35 | 30 | 25 |
| Light* | 0 | 3 | 5 | 5 | 4 | 0 |

**Winter (November-January) simulation of typical air temperatures, relative light intensitie**

| Time | 00:00 – 8:00 | 8:00 – 10:00 | 10:00 - 12:00 | 12:00 – 16:00 | 16:00 - 19:00 | 19:00 - 24:00 |
|---|---|---|---|---|---|---|
| Temperature | 15 | 15 | 20 | 25 | 20 | 15 |
| Light* | 0 | 3 | 5 | 5 | 4 | 0 |

*White light intensities correspond to (5) 100, (4) 70, (3) 30 μmol $m^{-2}$ $s^{-1}$, (0) designates darkness

**Summer simulation of seed bank extreme possible surface temperatures**

| Time | 00:00 – 6:00 | 6:00 – 9:00 | 9:00 - 12:00 | 12:00 – 17:00 | 17:00 - 19:00 | 19:00 - 24:00 |
|---|---|---|---|---|---|---|
| Temperature | 15 | 25 | 35 | 50 | 35 | 25 |
| Light* | 0 | 3 | 5 | 5 | 4 | 0 |

**Winter simulation of seed bank extreme possible surface temperatures**

| Time | 00:00 – 8:00 | 8:00 – 10:00 | 10:00 - 12:00 | 12:00 – 16:00 | 16:00 - 19:00 | 19:00 - 24:00 |
|---|---|---|---|---|---|---|
| Temperature | 4 | 15 | 20 | 25 | 15 | 10 |
| Light* | 0 | 3 | 5 | 5 | 4 | 0 |

*White light intensities correspond to (5) 100, (4) 70, (3) 30 μmol $m^{-2}$ $s^{-1}$, (0) designates darkness

### Species, seed collection sites and habitats:

■ *Raphanus raphanistrum* (RR)
Ra'anana (RAN) - N32º11'27.80'' E34º50'45.73''
RR distribution range in Israel (species is cosmopolitan)

▲ *Raphanus pugioniformis* (RP)
Yehudia (YHD) - N32º57'10.01'' E34º42'23.02''
RP distribution range in Israel (species is endemic)

*Continues next page →*

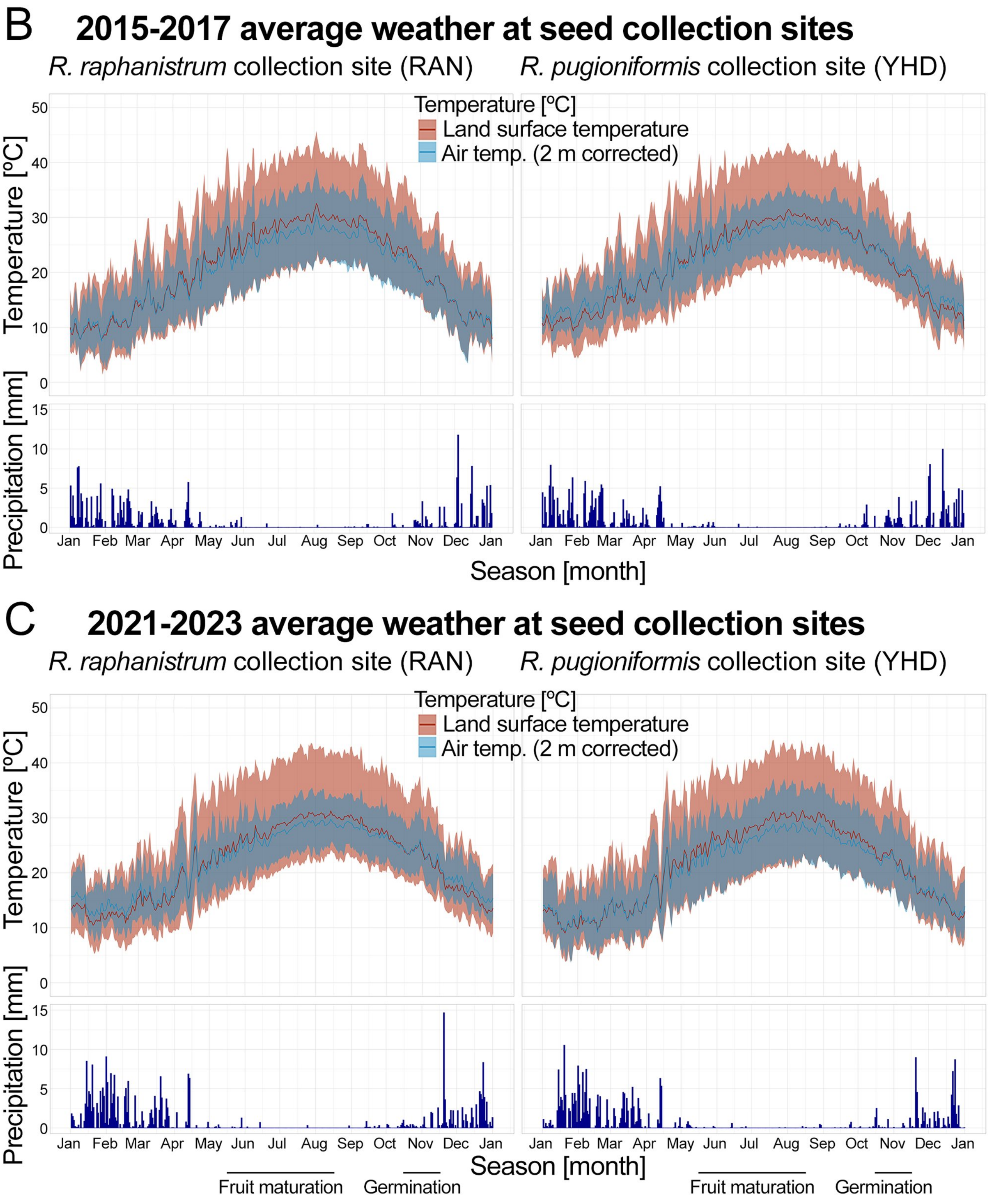


**Supplementary Fig. S1.** Seasonal weather and simulated weather of the wild radishes' native habitats. **(A)** The natural habitats and collection sites of *Raphanus raphanistrum* (RR, cosmopolitan) accession (RAN) and *Raphanus pugioniformis* (RP, endemic) accession (YHD) used in this experimental work and our published work [1-3]. **(B)** Seasonal weather data (2015-2017) for the RAN and YHD seed collection sites in Israel. Weather data was provided by Meteoblue (Basel, Switzerland) using their history+ product which uses

nonhydrostatic mesoscale modelling algorithms to generate localized historic meteorological data. Note that RR and RP fruits mature during the dry and hot summer months and germinate in late autumn. Fruits or fruit segments may also stay in the seed bank for several years until they germinate in late autumn. **(C)** Seasonal weather data (2015-2017) for the RAN and YHD seed collection sites in Israel. **(D)** Simulation of extreme weather of the natural habitat. Standard condition germination assays used 25ºC 16 h light, 15ºC 8 h darkness to mimick typical late autumn conditions. To mimic seed bank extreme conditions during summer (Figure 4D), possible land surface temperatures can reach >45ºC during the day.

**References:** [1] Bhattacharya S., Grone F., Przesdzink F., Ziffer-Berger J., Barazani O., Mummenhoff K., Kappert N. 2022 'Root of all success': Plasticity in root architecture of invasive wild radish for adaptive benefit. Front Plant Sci 13, 1035089. (doi:10.3389/fpls.2022.1035089). [2] Ziffer-Berger J., Waitz Y., Behar E., Ben Joseph O., Bezalel L., Wasserstrom H., Bajpai P.K., Bhattacharya S., Przesdzink F., Westberg E., et al. 2020 Seed dispersal of wild radishes and its association with within-population spatial distribution. BMC Ecol 20(1), 30. (doi:10.1186/s12898-020-00297-4). [3] Wasserstrom H., Ziffer-Berger J., Barzilai M., Mummenhoff K., Barazani O. 2022 Phenotypic variation of wild radishes *Raphanus pugioniformis* and *R. raphanistrum* associated with local conditions in the southeast Mediterranean. Flora 287, 151997. (doi:10.1016/j.flora.2021.151997).

A ***Raphanus raphanistrum*** **(RR)**

RR seedlot with fully-developed (FD) and ca. 18% under-developed (UD) seeds HSW UD 91.0 mg

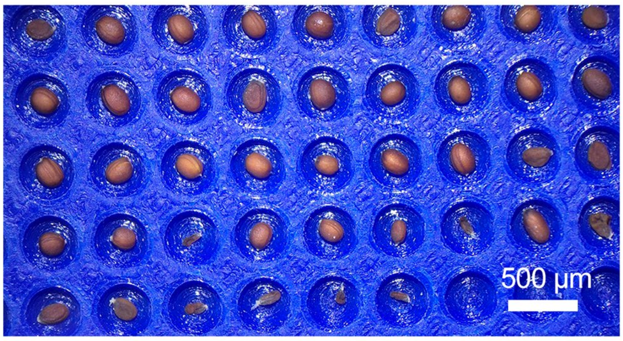


Cleaned RR seedlot with 100% FD seeds, Hundred Seed Weight (HSW) 318.3 mg

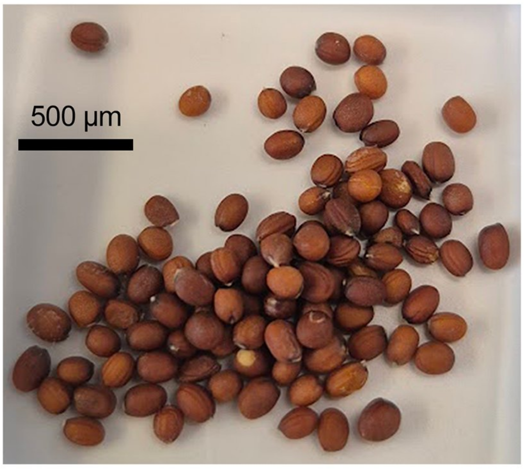


B ***Raphanus pugioniformis*** **(RP)**

RP HSW 650.0 mg

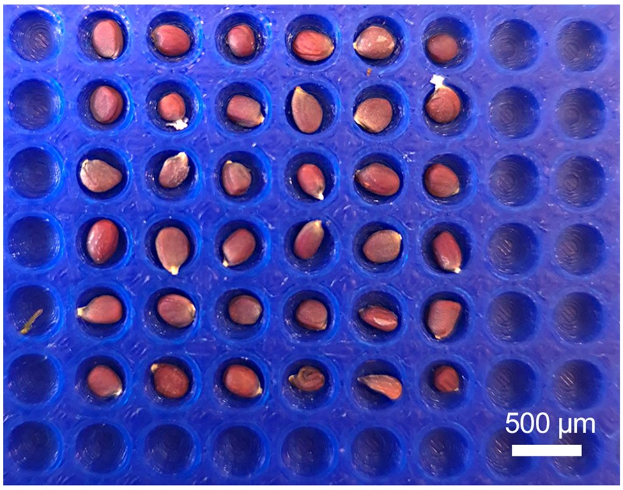


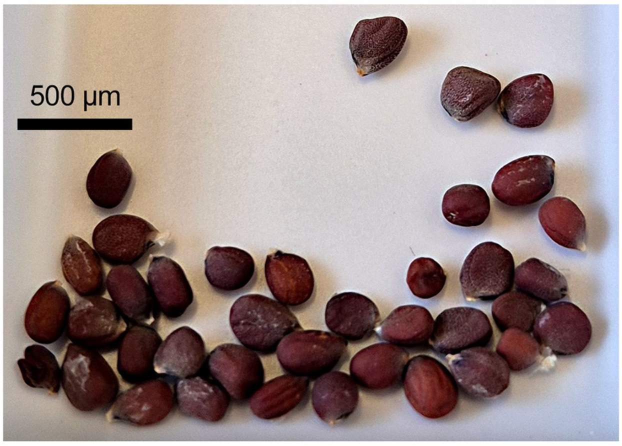


C

| **Species** | **Seed area [$mm^2$]** | | | | **Seed lenght [mm]** | | | | **Seed width [mm]** | | | |
|---|---|---|---|---|---|---|---|---|---|---|---|---|
| | **Mean** | SD | Minimum | Maximum | **Mean** | SD | Minimum | Maximum | **Mean** | SD | Minimum | Maximum |
| ***Raphanus raphanistrum*** n = 116 (FD seeds) | 3.12 | 0.49 | 2.19 | 4.48 | 2.30 | 0.18 | 1.92 | 2.76 | 1.78 | 0.17 | 1.40 | 2.21 |
| ***Raphanus pugioniformis*** n = 106 | 5.27 | 1.20 | 2.85 | 8.68 | 2.93 | 0.35 | 2.04 | 3.73 | 2.34 | 0.33 | 1.55 | 3.11 |

**Supplementary Fig. S2.** Morphological properties of *Raphanus raphanistrum* (RR) and *Raphanus pugioniformis* (RP) seeds. **(A)** The RR RAN seeds containing 18% under-developed seeds was cleaned manually to provide the RR seedlot with 100% fully-developed (FD) seeds used in the experiments. Its Hundred Seed Weight (HSW) was 318.3 mg. (B) The HSW of the RP YHD seedlot was 650.0 mg. **(C)** RR and RP seed size parameter (area, length, width) of the seedlots used in this work.

**A Mature RR and RP fruit (F) and seed (S), and RR late stage midvalve region (MVR) / predetermined breaking zone (PBZ) (enlarged part of Figure 2*a*)**

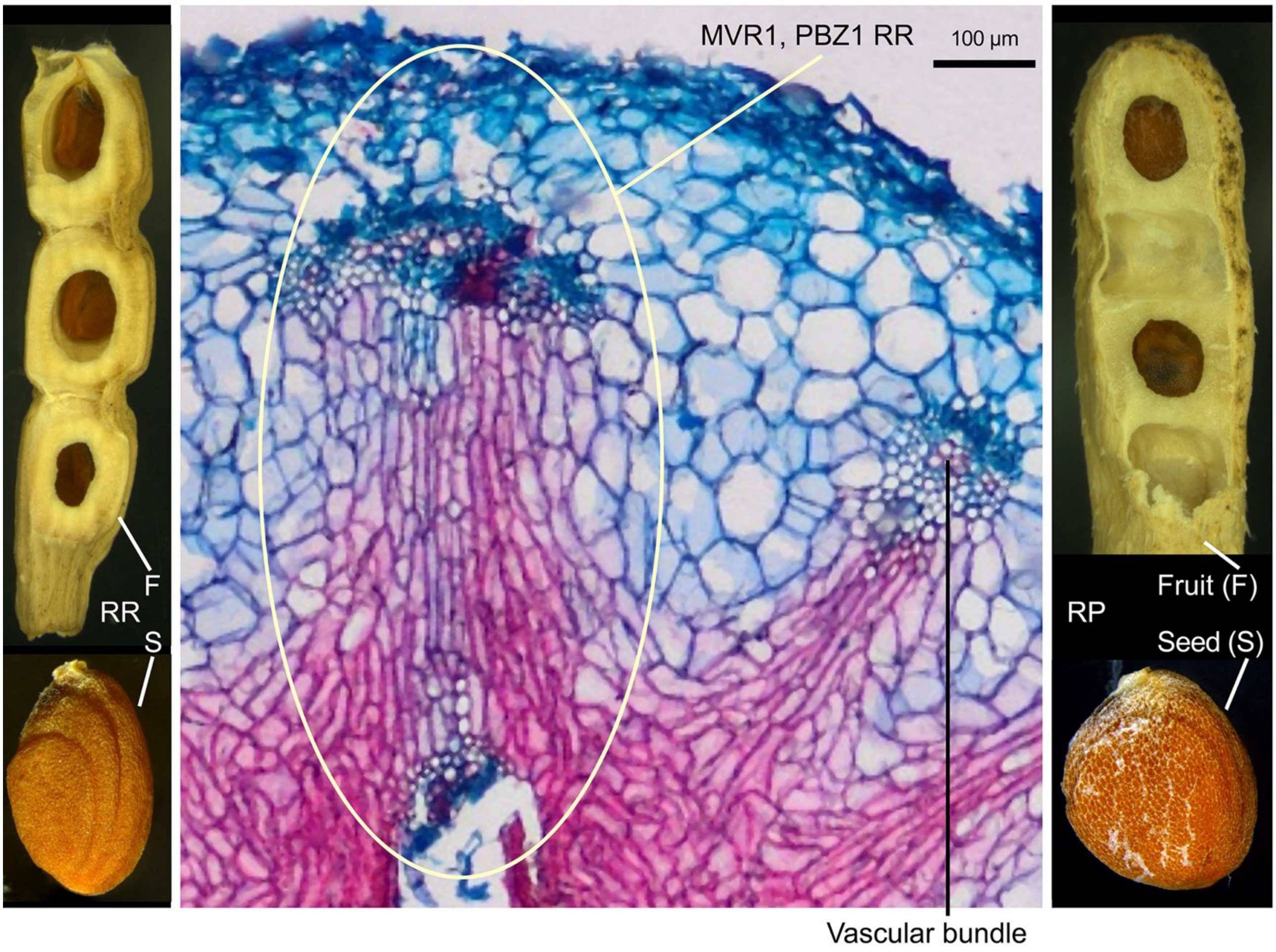


**B *Raphanus raphanistrum* (RR) fruit development, early and mid developmental stages**

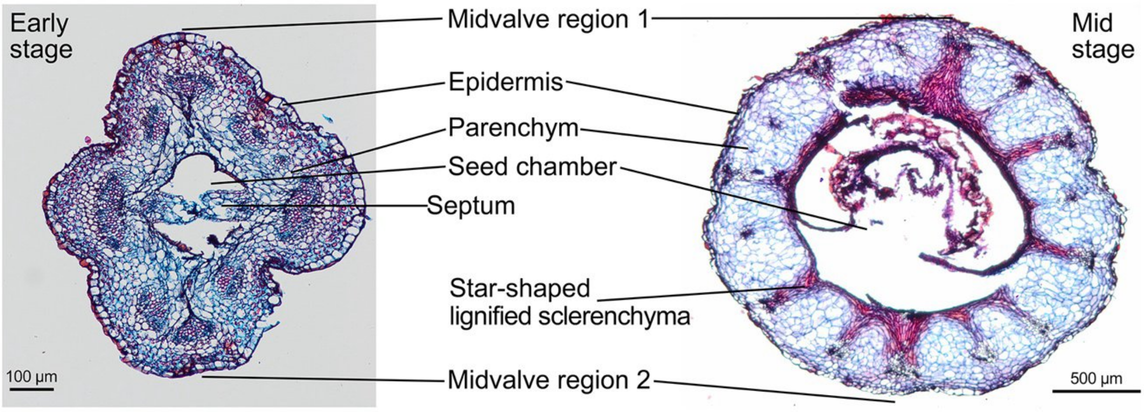


*Continues next page →*

## C Pericarp (longitudinal section) and seed of mature RR fruit

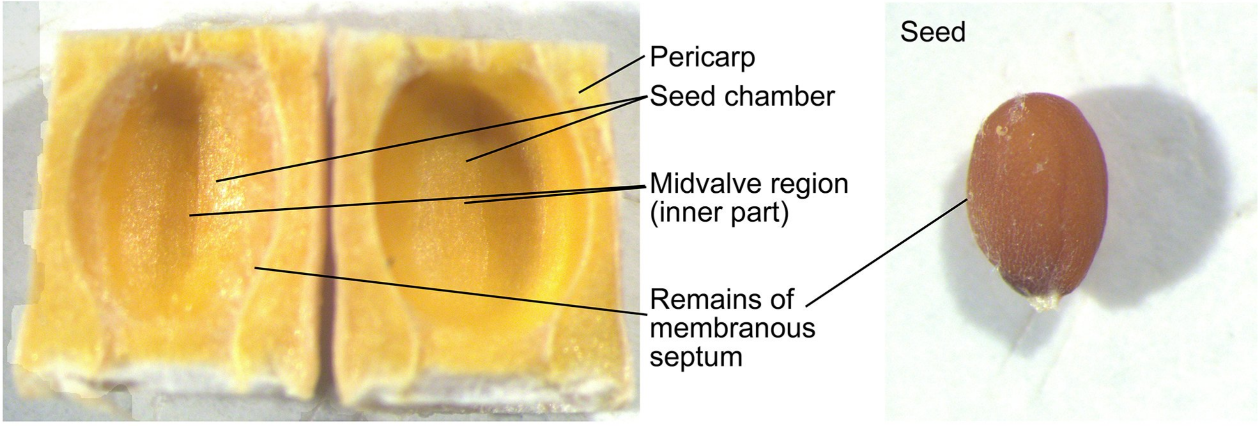


## D *Raphanus* spp. mature fruit morphology, septum and seed orientation

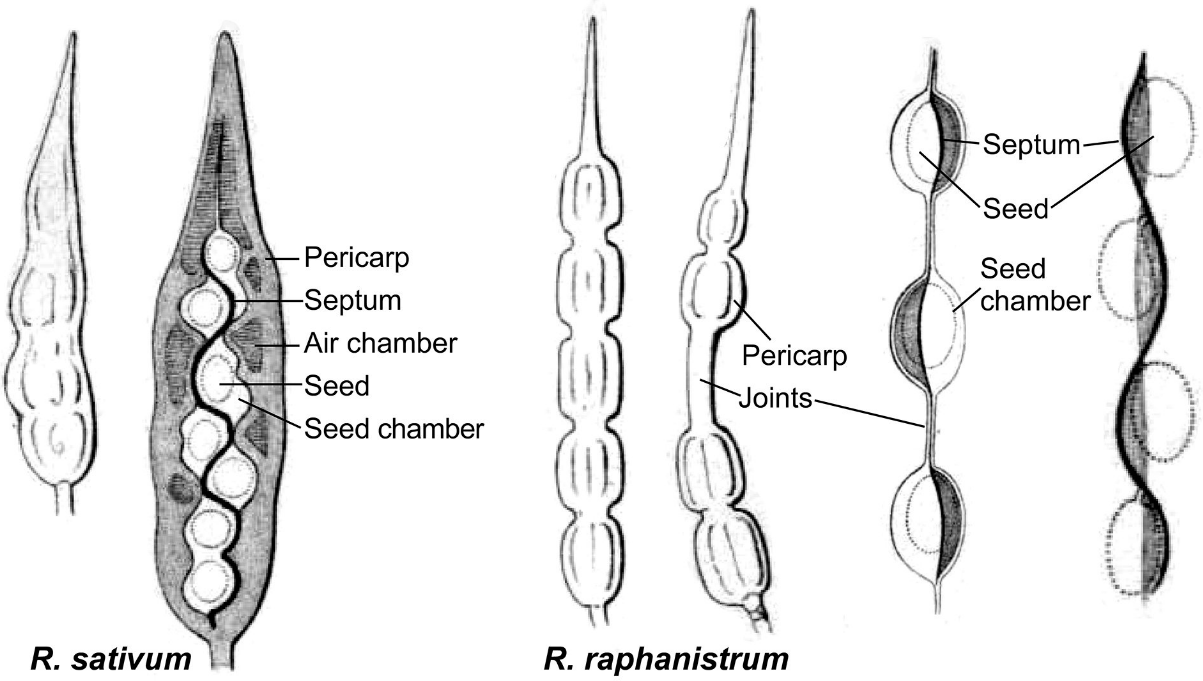


**Supplementary Fig. S3.** *Raphanus raphanistrum* (RR) fruit development and identification of the remains of the septum in mature *Raphanus* spp. fruits. **(A)** Mature RR and *R. pugioniformis* (RP) fruit cut open to show the seed chambers and seeds, and enlargement of the RR MVR1 from Figure 2A. **(B)** Early- and mid-stage RR fruit development with the position of the septum (early stage) being instrumental for defining the replum and midvalve regions (MVR). The presented early and mid RR stages correspond to *Arabidopsis thaliana* (Roeder A.H., Yanofsky M.F. 2006. Fruit development in Arabidopsis. Arabidopsis Book 4, e0075. doi:10.1199/tab.0075). fruit developmental stages 13 (anthesis) and 17/18 (fruit growth complete, yellowing starts), respectively. **(C)** Longitudional section of a mature fruit section in the non-MVR plane (*left*) and a mature seed (*right*). Note that a part of the inner seed chamber surface and the left half of the seed surface are covered with the remains of the membranous septum. **(D)** Drawings of *R. sativus* and *R. raphanistrum* mature fruits

including their internal morphology with the positioning of the seeds, seed chambers, septum and air chambers. In the lomentoid fruits of *Raphanus* species the almost invisible septum remains are pulling through the fruit in a wavelike manner. Drawings from a digital version of Hoffmann 1872, Botanische Zeitung 30, https://biodiversitylibrary.org/bibliography/4948, Missouri Botanical Garden.

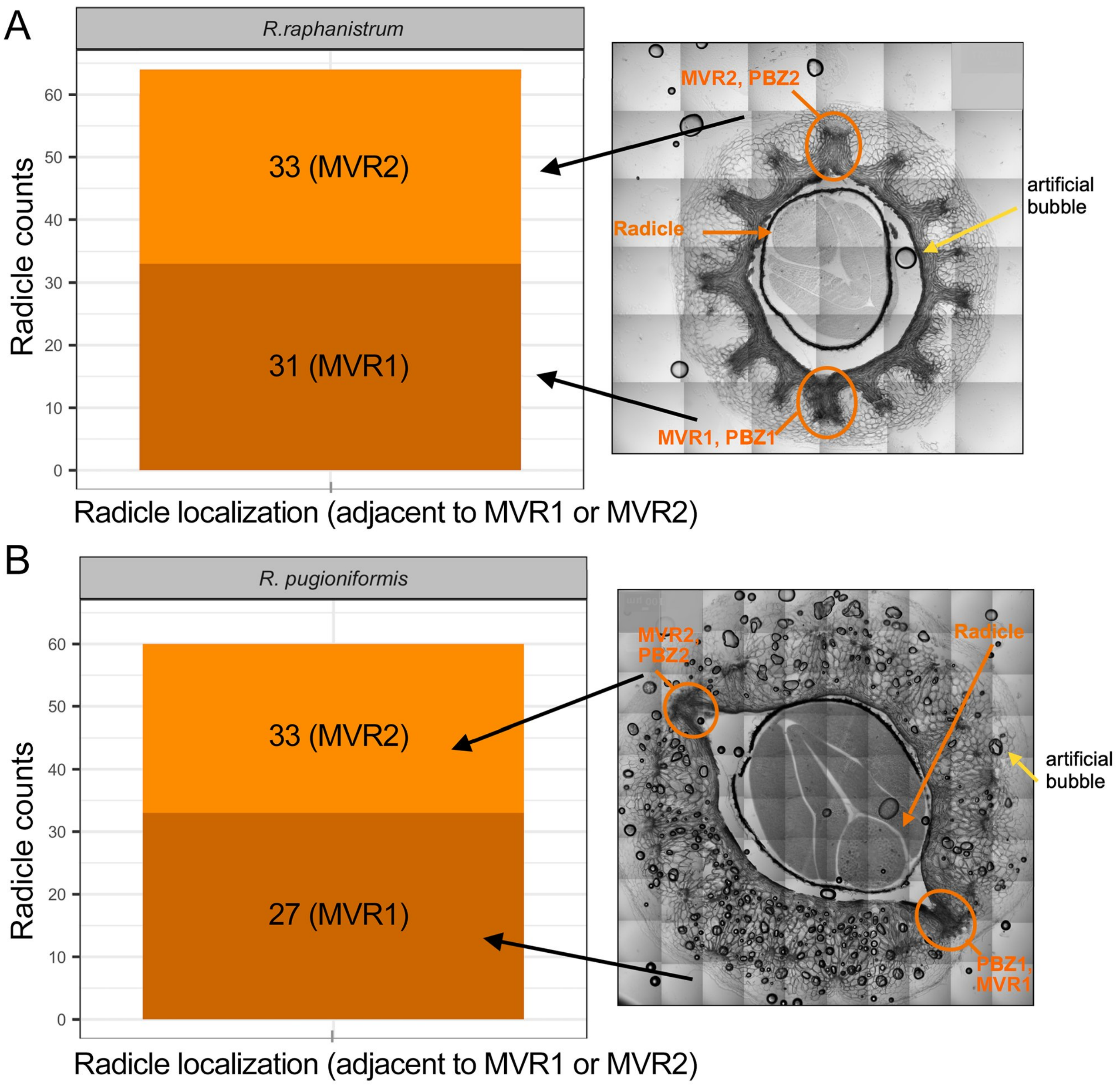


**Supplementary Fig. S4.** Frequency distribution of seed positioning within the seed chambers with the radicles adjacent to either midvalve region MVR1 or MVR2. **(A)** Frequency of *R. raphanistrum* radicle localization (*left panel*) derived from microscopic fruit sections (*right panel*). **(B)** Frequency of *Raphanus pugioniformis* radicle localization (*left panel*) derived from microscopic fruit sections (*right panel*). MVR1 and MVR2 contain the identified predetermined breaking zones PBZ1 and PBZ2, respectively. Note that there was no positioning preference for either MRV or PBZ. The radicles of the 64 RR seeds analysed, were 51.6% MVR2 and 48.4% MVR1 position; for the 60 RP seeds analysed this was 55.0% MVR2 and in 45.0% MVR1 position.

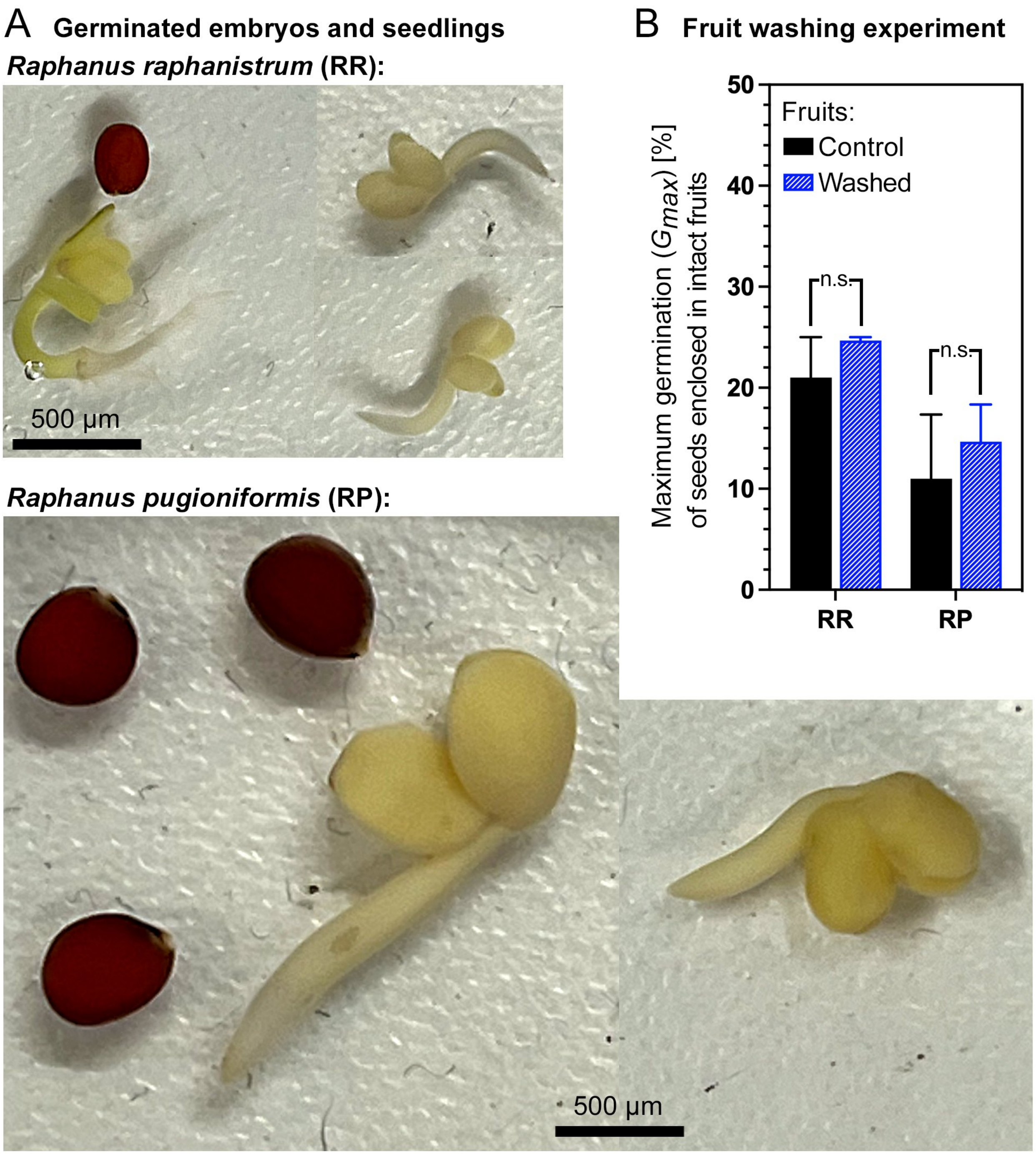


**Supplementary Fig. S5.** Germination and seedling growth of *Raphanus raphanistrum* (RR) and *Raphanus pugioniformis* (RP). **(A)** One-week old seedlings derived from bare seeds germinated in darkness in $dH_20$. These seedlings have stopped growing after seed reserves were exhausted and correspond to the late water uptake phase III in Figure 3C,D. **(B)** The effect of washing (24 h in $dH_2O$) intact RR and RP fruits on the maximum germination percentages ($G_{max}$) of RR and RP seeds enclosed in intact fruits under standard germination conditions (25ºC 16 h light, 15ºC 8 h darkness). Mean values ± SEM are presented for 3 fruits for each treatment containing an average of 5 (RR) and 3 (RP) seeds per fruit. Paired *t*-tests revealed no significant (n.s.) statistical differences.
